# Supplementary material for: The ENIGMA MEG Pipeline: Automated cortically localized spectral analysis of multi-site resting state MEG datasets
Source: bioRxiv. 2026 Jun 8:2026.06.03.729876. Preprint. [Version 1] doi: 10.64898/2026.06.03.729876 (PMC13277920; doi:10.64898/2026.06.03.729876)

Supplementary Tables 1-4: Confusion matrices from MEGnet retraining grouped by site/MEG vendor

	**Predicted Class - HCP**						
**Actual Class**		Non-Artifact	Blink	Cardiac	Saccade	**Sensitivity**	**False Negative Rate**
	Non-Artifact	517	2	9	0	97.92%	2.08%
	Blink	0	32	0	0	100.00%	0.00%
	Cardiac	0	0	39	0	100.00%	0.00%
	Saccade	0	0	0	21	100.00%	0.00%
	**Positive Predictive Value**	100.00%	94.12%	81.25%	100.00%	**Accuracy**	98.23%
	**False Discovery Rate**	0.00%	5.88%	18.75%	0.00%	**Error Rate**	1.77%

	**Predicted Class - NYU**						
**Actual Class**		Non-Artifact	Blink	Cardiac	Saccade	**Sensitivity**	**False Negative Rate**
	Non-Artifact	168	0	3	1	97.67%	2.33%
	Blink	0	10	0	0	100.00%	0.00%
	Cardiac	0	0	14	0	100.00%	0.00%
	Saccade	0	0	0	4	100.00%	0.00%
	**Positive Predictive Value**	100.00%	100.00%	82.35%	80.00%	**Accuracy**	98.00%
	**False Discovery Rate**	0.00%	0.00%	17.65%	20.00%	**Error Rate**	2.00%

	Predicted Class - CAMCAN						
**Actual Class**		Non-Artifact	Blink	Cardiac	Saccade	**Sensitivity**	**False Negative Rate**
	Non-Artifact	1040	2	7	2	98.95%	1.05%
	Blink	0	58	0	0	100.00%	0.00%
	Cardiac	0	0	56	0	100.00%	0.00%
	Saccade	0	0	0	15	100.00%	0.00%
	**Positive Predictive Value**	100.00%	96.67%	88.89%	88.24%	**Accuracy**	99.07%
	**False Discovery Rate**	0.00%	3.33%	11.11%	11.76%	**Error Rate**	0.93%

	Predicted Class - NIH						
**Actual Class**		Non-Artifact	Blink	Cardiac	Saccade	**Sensitivity**	**False Negative Rate**
	Non-Artifact	1460	5	9	5	98.72%	1.28%
	Blink	0	79	0	0	100.00%	0.00%
	Cardiac	0	0	86	0	100.00%	0.00%
	Saccade	0	0	0	36	100.00%	0.00%
	**Positive Predictive Value**	100.00%	94.05%	90.53%	87.80%	**Accuracy**	98.87%
	**False Discovery Rate**	0.00%	5.95%	9.47%	12.20%	**Error Rate**	1.13%

Supplementary Tables 5-7: Confusion matrices from MEGnet retraining grouped by eyes open rest, eyes closed rest, and task

	Predicted Class - Rest Eyes Open						
**Actual Class**		Non-Artifact	Blink	Cardiac	Saccade	**Sensitivity**	**False Negative Rate**
	Non-Artifact	393	0	6	1	98.25%	1.75%
	Blink	0	23	0	0	100.00%	0.00%
	Cardiac	0	0	30	0	100.00%	0.00%
	Saccade	0	0	0	7	100.00%	0.00%
	**Positive Predictive Value**	100.00%	100.00%	83.33%	87.50%	**Accuracy**	98.48%
	**False Discovery Rate**	0.00%	0.00%	16.67%	12.50%	**Error Rate**	1.52%

	Predicted Class - Rest Eyes Closed						
**Actual Class**		Non-Artifact	Blink	Cardiac	Saccade	**Sensitivity**	**False Negative Rate**
	Non-Artifact	386	0	2	0	99.48%	0.52%
	Blink	0	19	0	0	100.00%	0.00%
	Cardiac	0	0	24	0	100.00%	0.00%
	Saccade	0	0	0	9	100.00%	0.00%
	**Positive Predictive Value**	100.00%	100.00%	92.31%	100.00%	**Accuracy**	99.55%
	**False Discovery Rate**	0.00%	0.00%	7.69%	0.00%	**Error Rate**	0.45%

	Predicted Class -Task						
**Actual Class**		Non-Artifact	Blink	Cardiac	Saccade	**Sensitivity**	**False Negative Rate**
	Non-Artifact	2406	9	20	7	98.53%	1.47%
	Blink	0	137	0	0	100.00%	0.00%
	Cardiac	0	0	141	0	100.00%	0.00%
	Saccade	0	0	0	60	100.00%	0.00%
	**Positive Predictive Value**	100.00%	93.84%	87.58%	89.55%	**Accuracy**	98.71%
	**False Discovery Rate**	0.00%	6.16%	12.42%	10.45%	**Error Rate**	1.29%

Supplementary Figure 1: Distribution of ages for the three cohort datasets.

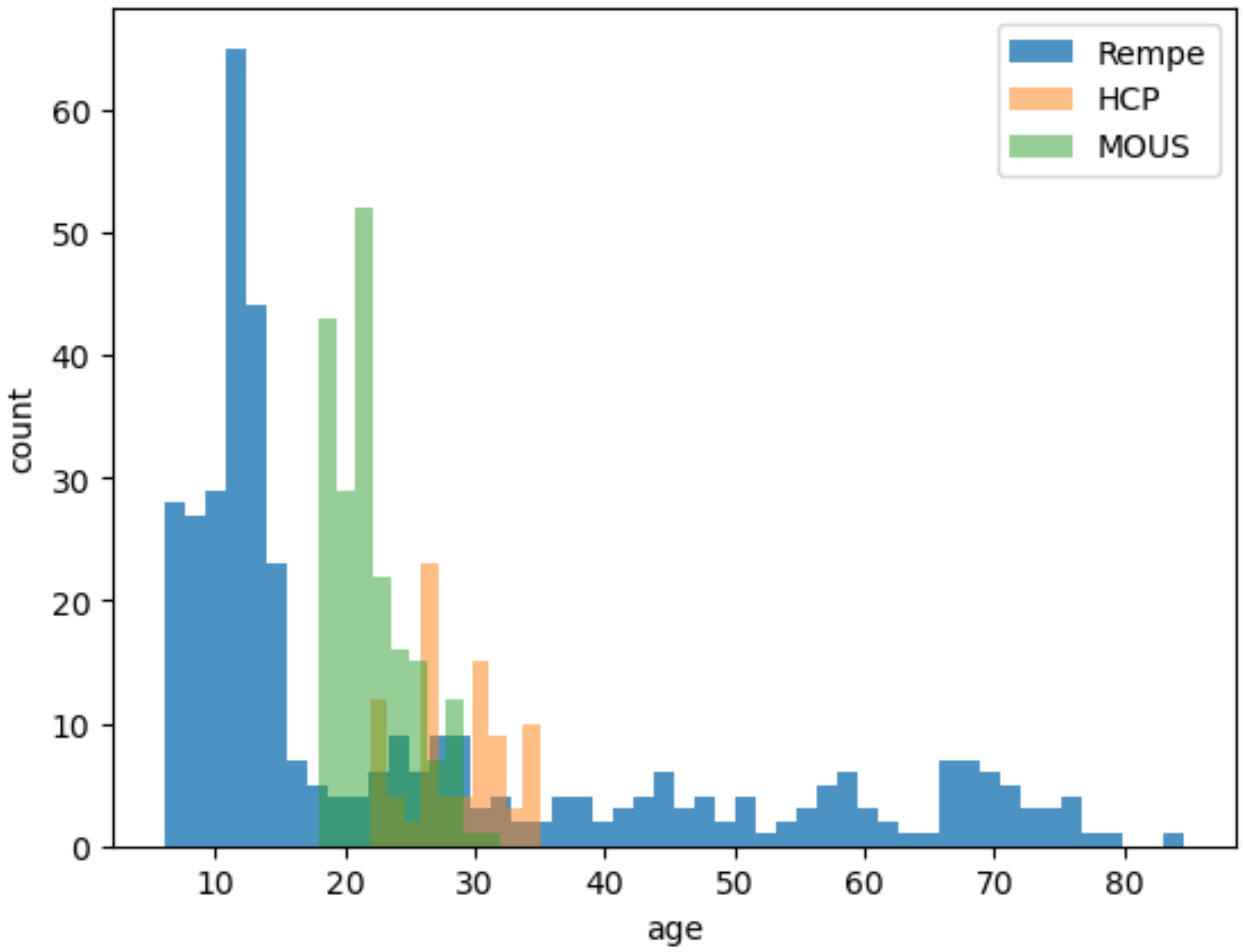

Supplement: Supplementary file 1 [file media-1.pdf]
